# Comparative effectiveness of tirzepatide versus thiazolidinedione in adults with MASLD: a propensity score-matched cohort study

**DOI:** 10.3389/fphar.2026.1769425

**Published:** 2026-05-15

**Authors:** Jheng-Yan Wu, Yu-Min Lin, Wan-Hsuan Hsu, Ting-Hui Liu, Ya-Wen Tsai, Po-Yu Huang, Min-Hsiang Chuang, Chih-Cheng Lai

**Affiliations:** 1 Department of Nutrition, Chi Mei Medical Center, Tainan, Taiwan; 2 Department of Public Health, College of Medicine, National Cheng Kung University, Tainan, Taiwan; 3 Division of Cardiology, Department of Internal Medicine, Chi Mei Medical Center, Tainan, Taiwan; 4 Department of Internal Medicine, Chi Mei Medical Center, Tainan, Taiwan; 5 Department of Psychiatry, Chi Mei Medical Center, Tainan, Taiwan; 6 Division of Preventive Medicine, Chi Mei Medical Center, Tainan, Taiwan; 7 Department of Intensive Care Medicine, Chi Mei Medical Center, Tainan, Taiwan; 8 School of Medicine, College of Medicine, National Sun Yat-sen University, Kaohsiung, Taiwan

**Keywords:** diabetes, MASLD, outcomes, thiazolidinedione, tirzepatide

## Abstract

**Background:**

Tirzepatide (TZP) and thiazolidinediones (TZDs) have both shown promise in treating metabolic dysfunction-associated steatotic liver disease (MASLD), yet direct comparative evidence remains scarce. This real-world study aimed to compare the clinical effectiveness of TZP and TZDs in adults diagnosed with MASLD.

**Methods:**

We conducted a retrospective, multi-institutional cohort study using data from the TriNetX global health research network. Adults with MASLD who were newly initiated on TZP or TZD were included. The primary outcome was a composite measure encompassing all-cause mortality, major adverse liver outcome (MALO), major adverse cardiovascular event (MACE), and major adverse kidney event (MAKE). Hazard ratios (HRs) and 95% confidence intervals (CIs) were calculated using Cox proportional hazards models.

**Results:**

After 1:1 propensity score matching, each treatment arm included 9,262 patients. TZP use was significantly associated with a reduced risk of the composite primary outcome compared to TZD (HR, 0.66; 95% CI, 0.58–0.42). Additionally, TZP was linked to lower risks of all-cause mortality (HR, 0.48; 95% CI, 0.32–0.71), MALO (HR, 0.50; 95% CI, 0.36–0.69), MACE (HR, 0.65; 95% CI, 0.51–0.82), and MAKE (HR, 0.50; 95% CI, 0.36–0.69). These associations were consistent across subgroups stratified by age, sex, body mass index, and underlying comorbidities.

**Conclusion:**

In this large real-world cohort study, TZP was associated with lower risks of all-cause mortality, MALO, MACE, and MAKE compared to TZDs in adults with MASLD. While these findings suggest a potential clinical advantage, the observational design precludes causal inference. Prospective randomized trials are needed to confirm these associations and establish evidence-based treatment recommendations.

## Introduction

Metabolic dysfunction-associated steatotic liver disease (MASLD), previously referred to as non-alcoholic fatty liver disease (NAFLD), represents a significant and growing global health challenge, affecting an estimated 25%–30% of the adult population worldwide (Younossi et al., 2025; European Association for the Study of the Liver, 2024). Projections suggest that by 2040, the global prevalence of MASLD among adults will exceed 55% (Younossi et al., 2025). MASLD comprises a continuum of liver conditions ranging from simple steatosis to metabolic dysfunction-associated steatohepatitis (MASH), with potential progression to cirrhosis and hepatocellular carcinoma (HCC) (Owrangi et al., 2025). The condition is strongly associated with metabolic syndrome components, including obesity, T2D, dyslipidemia, and hypertension. In addition to liver-related morbidity, patients with MASLD face heightened risks of cardiovascular mortality, chronic kidney disease, and extrahepatic malignancies, all of which contribute to significant healthcare costs (Zhou et al., 2024; Ciardullo et al., 2024; Chan et al., 2024).

Considering its rising global burden and serious clinical consequences, there is an urgent need for effective pharmacologic treatments for MASLD. Thiazolidinediones (TZDs) have demonstrated benefits in several randomized controlled trials (RCTs) (Lee et al., 2025; Ratziu et al., 2008; Promrat et al., 2004) and meta-analyses (Mahady et al., 2011; Musso et al., 2017) showing improvements in liver histology, including reductions in steatosis, inflammation, and fibrosis. These findings have supported the use of TZDs to slow disease progression in patients with MASLD or MASH. Current guidelines recommend TZDs, along with sodium-glucose cotransporter 2 inhibitors (SGLT2is) and glucagon-like peptide-1 receptor agonists (GLP-1RAs), for patients with MASLD and T2D (Diaz et al., 2025; American Diabetes Association Professional Practice Committee, 2025). However, the clinical use of TZDs may be constrained by adverse effects such as weight gain, fluid retention, and an elevated risk of heart failure (Tang et al., 2003; Karalliedde and Buckingham, 2007).

To address these limitations, attention has turned to newer agents with more favorable safety and metabolic profiles. Tirzepatide (TZP), a dual glucose-dependent insulinotropic polypeptide (GIP) and GLP-1 receptor agonist, has shown considerable promise. In addition to its glucose-lowering effects, TZP induces substantial weight loss and may offer cardiovascular and heart failure benefits (Karalliedde and Buckingham, 2007; Jastreboff et al., 2022; Lin et al., 2025). SYNERGY-NASH trial demonstrated that TZP significantly improved MASH resolution without worsening fibrosis when compared to placebo (Loomba et al., 2024). Moreover, nearly half of the patients treated with TZP achieved at least a one-stage improvement in fibrosis without worsening MASH, compared to 30% in the placebo group (Loomba et al., 2024).

Despite the established benefits of TZDs in MASLD and the emerging promise of tirzepatide demonstrated in the SYNERGY-NASH trial (Loomba et al., 2024), Prior network meta-analyses suggest that GLP-1RA outperform TZDs in reducing liver fat content, BMI, and waist circumference in overweight or obese patients with MASLD outcomes (Park et al., 2023). However, whether TZP’s dual GIP/GLP-1 receptor agonism translates into superior real-world effectiveness over TZDs across the broader spectrum of MASLD outcomes, including liver-related, cardiovascular, and renal endpoints, remains unknown. To fill this important knowledge gap, we conducted a retrospective cohort study using a large real-world database to evaluate the comparative effectiveness of TZP versus TZD in adults diagnosed with MASLD.

## Methods

### Data source

This retrospective cohort study utilized data from the TriNetX research network, a global federated platform comprising electronic health records from approximately 170 million individuals from 146 healthcare organizations (HCOs) across North America, Europe, the Middle East, and the Asia-Pacific region. The dataset includes comprehensive clinical information such as diagnoses, prescriptions, laboratory findings, procedures, and genomic profiles. The Chi Mei Hospital institutional review board approved this TriNetX database study (approval number: 11402-E02) and waived the requirement for informed consent since the research used only aggregated statistical data from de-identified sources. All study procedures were conducted in accordance with the Strengthening the Reporting of Observational Studies in Epidemiology (STROBE) guidelines.

### Study design

We identified adult individuals (≥18 years) with a diagnosis of MASLD who initiated either TZP or TZD therapy between 1 January 2022, and 31 May 2025. MASLD was defined based on the presence of hepatic steatosis, determined by diagnostic codes for NAFLD or nonalcoholic steatohepatitis (NASH), in combination with at least one cardiometabolic risk factor. These included insulin resistance (evidenced by a diagnosis of T2D, hemoglobin A1c ≥ 5.7%, use of antidiabetic agents, or fasting plasma glucose ≥100 mg/dL), central obesity (body mass index [BMI] ≥ 25 kg/m^2^ or waist circumference ≥94 cm), elevated blood pressure (systolic blood pressure [SBP] ≥ 130 mmHg, diastolic blood pressure ≥85 mmHg, or use of antihypertensives), reduced high-density lipoprotein cholesterol (HDL <40 mg/dL or the use of lipid-modifying therapy), and hypertriglyceridemia (triglyceride concentration ≥150 mg/dL) (Kuo et al., 2025a; Kuo et al., 2025b).

Participants were categorized into two groups based on treatment exposure. The TZP group included individuals who initiated TZP following a diagnosis of MASLD, whereas the TZD group consisted of those who commenced TZD therapy after their MASLD diagnosis. The index date was defined as the date of the first prescription of the assigned study drug. To preserve the integrity of the new-user design and avoid misclassification bias, individuals were excluded if they: (1) had prior exposure to the assigned treatment before the index date; (2) experienced any study outcomes, including major adverse cardiovascular events (MACEs), major adverse kidney events (MAKEs), or major adverse liver outcomes (MALOs) before the index date; (3) received the comparator drug at any time prior to the index date; or (4) had insufficient follow-up information. A detailed description of all definitions and coding algorithms used to identify baseline characteristics, clinical conditions, medications, procedures, and laboratory values is provided in Supplementary Table 1.

### Covariates and propensity score matching

After defining the study groups, index dates, outcomes, and relevant covariates, we constructed a baseline covariate matrix using data collected during the 12-month period preceding each patient’s index date. Propensity scores were estimated using logistic regression models, which calculated the likelihood of assignment to the TZD group based on observed baseline characteristics. We implemented 1:1 matching between groups using a greedy nearest-neighbor algorithm with a caliper of 0.1 pooled standard deviations, ensuring optimal alignment between individuals in the smaller and larger cohorts. Covariate balance between matched groups was assessed using standardized mean differences (SMD), with values below 0.1 indicating acceptable balance (Haukoos and Lewis, 2015).

The propensity score matching (PSM) procedure accounted for a comprehensive range of baseline covariates. Demographic variables included age (years), sex (female or male), and race (White, Black or African American, Asian, Other, or Unknown). Clinical comorbidities considered in the matching included T2D, dyslipidemia, overweight and obesity, chronic kidney disease, nicotine dependence, alcohol-related disorders, chronic lower respiratory diseases, ischemic heart diseases, cerebrovascular diseases, heart failure, atrial fibrillation and flutter, obstructive sleep apnea, neoplasms, fatty liver, hepatic fibrosis, MASH, and cirrhosis. T2D-related complications were classified into kidney, ophthalmic, neurological, and circulatory categories. Medication use was included in the covariates and covered both antihypertensive agents, including angiotensin-converting enzyme inhibitors (ACEis), angiotensin II receptor blockers (ARBs), beta-blockers, calcium channel blockers, and diuretics, and lipid-lowering agents, such as HMG-CoA reductase inhibitors, fibrates, and ezetimibe. Anti-diabetic drug use comprised metformin, sulfonylureas, thiazolidinediones, alpha-glucosidase inhibitors, dipeptidyl peptidase-4 inhibitors (DPP4is), SGLT2is, GLP-1RAs, and insulin. In addition, the analysis incorporated key laboratory and clinical parameters, including aspartate aminotransferase, alanine aminotransferase, platelet counts, hemoglobin A1c level, estimated glomerular filtration rate, BMI, HDL-C, low-density lipoprotein cholesterol, total cholesterol, and triglycerides. All variables were evaluated before and after matching to ensure balance between the TZP and TZD groups. Complete definitions and operational codes for all covariates are provided in Supplementary Table 2.

### Outcomes and follow-up

The primary outcome was a composite endpoint encompassing all-cause mortality, MACEs, MAKEs, and MALOs. Each of these individual components also served as predefined secondary outcomes, as defined in previous studies (Wu et al., 2025a; Wu et al., 2025b; Wu et al., 2025c). Briefly, MACEs were defined as the incidence of cerebral infarction, acute myocardial infarction, cardiac arrest, or death. MAKEs included the onset of end-stage renal disease, resumption of dialysis, initiation of new dialysis treatment, or death. MALOs were defined by the presence of complications such as bleeding esophageal varices, hepatic encephalopathy, ascites-related events, hepatocellular carcinoma, liver transplantation, or death. In addition to effectiveness outcomes, selected safety outcomes were also evaluated, including nausea, vomiting, diarrhea, edema, heart failure exacerbation, fracture, and hypoglycemia. Outcome assessment began 1 day after the index date and continued until the first occurrence of an outcome event, patient death, the last recorded clinical visit, or 12 months post-index date, whichever came first.

### Statistical analysis

Continuous variables were reported as means with standard deviations, while categorical variables were presented as frequencies and percentages. To enhance comparability across treatment groups, PSM was applied prior to conducting primary, subgroup, and sensitivity analyses to balance baseline covariates. Time-to-event outcomes were evaluated using Cox proportional hazards regression to estimate hazard ratios (HRs) and corresponding 95% confidence intervals (CIs). Kaplan-Meier plots and log-rank tests were employed to compare survival distributions between groups. Prespecified subgroup analyses were conducted stratified by age, sex, BMI, TZD types, and comorbid conditions including type 2 diabetes, heart failure, obstructive sleep apnea, MASH, and cirrhosis. To examine the validity and robustness of our results, we selected negative control outcomes, including hernia, hearing loss, and traumatic brain injury, based on their presumed lack of pharmacological association with TZP or TZD. Hernias are mechanical structural conditions unlikely to be influenced by TZP or TZD; hearing loss represents a sensory disorder with no established metabolic drug pathway; and traumatic brain injury is an externally caused event biologically independent of the study medications. Additionally, we calculated E-values to evaluate the potential influence of residual confounding (VanderWeele and Ding, 2017). A landmark analysis was also implemented to explore the impact of time-dependent effects (Morgan, 2019). All statistical procedures were executed within the TriNetX analytics platform.

### Additional analysis

To further evaluate potential confounding related to prior GLP-1RA exposure, we performed a sensitivity analysis restricted to patients without prior GLP-1RA use before the index date. We also conducted a sensitivity analysis extending the follow-up period to 2 years to assess whether the observed associations persisted over a longer observation period. Additionally, we performed a sensitivity analysis examining the individual components of the composite outcome separately, to better distinguish mortality from non-fatal clinical events.

## Results

### Patients’ selection

**FIGURE 1:**
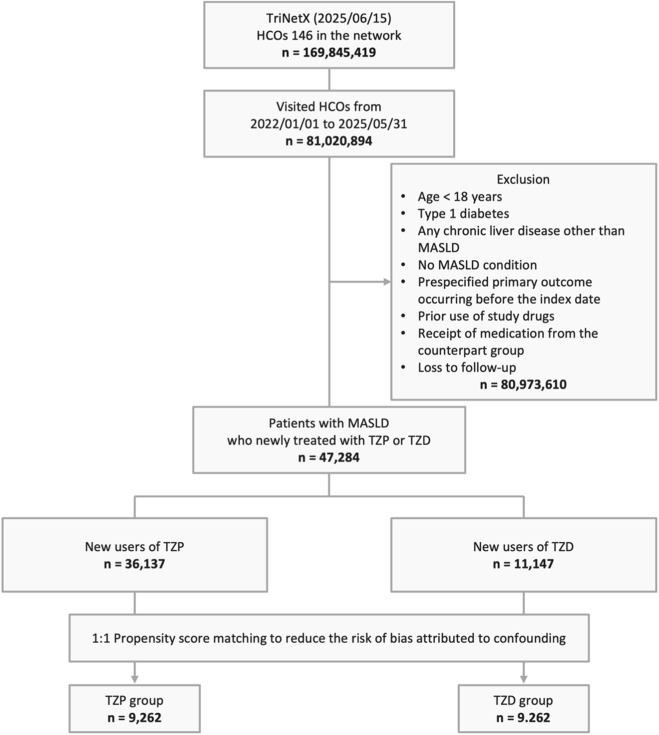
Study design and selection flow. HCO, healthcare organization; MASLD, metabolic dysfunction-associated steatotic liver disease; TZD, thiazolidinedione; TZP, tirzepatide.

A total of 169,845,419 adults were identified from 146 HCOs within the Global Collaborative Network of the TriNetX platform on 15 June 2025. Of these, 81,020,894 had records of HCO visits between 1 January 2022, and 31 May 2025. After applying exclusion criteria, 47,284 patients with MASLD who were newly treated with TZP or TZD remained. Of these, 36,137 were newly prescribed TZP, while 11,147 received TZD. After 1:1 PSM to reduce the risk of bias attributed to confounding, 9,262 well-matched patients were included in both the TZP and TZD groups (Figure 1). The median follow-up duration was 289 days (168–365 days) in the TZP group and 365 days (365–365 days) in the TZD group.

### Demographic features of included patients

Before PSM, individuals in the TZP group were younger than those in the TZD group (55.5 ± 12.0 years vs. 59.4 ± 12.6 years), with significant differences in the distributions of sex and race. The TZP group had a higher prevalence of overweight/obesity (65.6% vs. 60.5%), chronic kidney disease (55.8% vs. 28.4%), ischemic heart diseases (21.6% vs. 14.7%), obstructive sleep apnea (30.4% vs. 13.0%), atrial fibrillation and flutter (7.6% vs. 3.7%), and neoplasms (5.7% vs. 3.9%) compared to the TZD group. Prior use of GLP-1RAs was more common in the TZP group (49.7% vs. 17.4%), while sulfonylureas (9.8% vs. 28.5%) and DPP4is (4.4% vs. 16.9%) were more common in the TZD group. The TZP group had higher BMI (38.6 ± 8.0 vs. 33.6 ± 7.5 kg/m^2^), lower proportion with HbA1c ≥ 7% (42.9% vs. 54.6%), lower proportion with HDL cholesterol <50 mg/dL (49.2% vs. 42.2%), and higher proportion with triglycerides ≥150 mg/dL (38.4% vs. 35.0%). After PSM, baseline characteristics were well balanced between the TZP and TZD groups, with SMDs <0.1 for all variables except BMI level (Table 1).

**TABLE 1:** Baseline characteristics of included subjects.

Variables	Before matching			After matching		
	TZP group (n = 36,137)	TZD group (n = 11,147)	SMD	TZP group (n = 9,262)	TZD group (n = 9,262)	SMD
Age at index, years						
Mean (SD)	55.5 (12.0)	59.4 (12.6)	0.314	58.3 (11.6)	58.6 (12.6)	0.019
Sex, n (%)						
Female	22,390 (62.0)	5,903 (53.1)	0.179	5,054 (54.6)	5,083 (54.9)	0.006
Male	12,820 (35.5)	5,027 (45.3)	0.200	4,049 (43.7)	4,017 (43.4)	0.007
Race, n (%)						
White	26,756 (74)	7,447 (67.0)	0.154	6,596 (71.2)	6,567 (70.9)	0.007
Black or african american	3,333 (9.2)	710 (6.4)	0.106	643 (6.9)	649 (7.0)	0.003
Asian	1,308 (3.6)	1,092 (9.8)	0.250	629 (6.8)	633 (6.8)	0.002
Other race	1,552 (4.3)	506 (4.6)	0.013	446 (4.8)	436 (4.7)	0.005
Unknown race	2,717 (7.5)	1,225 (11.0)	0.121	850 (9.2)	874 (9.4)	0.009
Comorbidities, n (%)						
T2D	29,913 (82.7)	9,252 (83.3)	0.015	7,529 (82.0)	7,508 (81.7)	0.006
Disorders of lipoprotein metabolism and other lipidemias	23,742 (65.7)	6,919 (62.3)	0.071	5,738 (62.0)	5,687 (61.4)	0.011
Overweight and obesity	23,702 (65.6)	6,723 (60.5)	0.105	5,527 (59.7)	5,576 (60.2)	0.011
Chronic kidney disease	20,163 (55.8)	3,151 (28.4)	0.578	2,986 (32.2)	2,989 (32.3)	0.001
Nicotine dependence	3,822 (10.6)	1,217 (11.0)	0.012	968 (10.5)	962 (10.4)	0.002
Chronic lower respiratory diseases	584 (1.6)	151 (1.4)	0.021	119 (1.3)	127 (1.4)	0.008
Ischemic heart diseases	7,823 (21.6)	1,630 (14.7)	0.182	1,441 (15.6)	1,440 (15.5)	<0.001
Cerebrovascular diseases	5,528 (15.3)	1,494 (13.5)	0.053	1,254 (13.5)	1,270 (13.7)	0.005
Heart failure	1,592 (4.4)	620 (5.6)	0.054	453 (4.9)	493 (5.3)	0.020
Atrial fibrillation and flutter	2,729 (7.6)	413 (3.7)	0.167	401 (4.3)	394 (4.3)	0.004
Obstructive sleep apnea	10,998 (30.4)	1,455 (13.0)	0.429	1,386 (15.4)	1,376 (15.3)	0.003
Neoplasms	2,065 (5.7)	433 (3.9)	0.085	384 (4.1)	390 (4.2)	0.003
MASH	2,653 (7.3)	772 (7.0)	0.015	630 (6.8)	650 (7.0)	0.009
Other and unspecified cirrhosis of liver	1,357 (3.8)	425 (3.8)	0.004	380 (4.1)	372 (4.0)	0.004
T2D related complication, n (%)						
Kidney complications	4,275 (11.8)	1,369 (12.3)	0.015	1,140 (12.3)	1,065 (11.5)	0.025
Ophthalmic complications	1,665 (4.6)	486 (4.4)	0.011	413 (4.5)	399 (4.3)	0.007
Neurological complications	4,528 (12.5)	1,364 (12.3)	0.008	1,154 (12.5)	1,113 (12.0)	0.014
Circulatory complications	2,075 (5.7)	477 (4.3)	0.066	408 (4.4)	415 (4.5)	0.004
Antihypertensives, n (%)						
ACEis	6,998 (19.4)	2,613 (23.5)	0.101	2,050 (22.1)	2,029 (21.9)	0.005
ARBs	9,056 (25.1)	2,258 (20.3)	0.113	1,856 (20)	1,862 (20.1)	0.002
Beta blockers	10,232 (28.3)	2,730 (24.6)	0.085	2,220 (24)	2,294 (24.8)	0.019
Calcium channel blockers	6,924 (19.2)	2,083 (18.8)	0.010	1,617 (17.5)	1,675 (18.1)	0.016
Diuretics	10,943 (30.3)	2,614 (23.5)	0.153	2,180 (23.5)	2,239 (24.2)	0.015
Lipid-lowering medication, n (%)						
HMG CoA reductase inhibitors	17,219 (47.6)	5,328 (48.0)	0.006	4,298 (46.4)	4,263 (46)	0.008
Fibrates	1,604 (4.4)	762 (6.9)	0.105	607 (6.6)	563 (6.1)	0.020
Ezetimibe	1,429 (4.0)	359 (3.2)	0.039	288 (3.1)	305 (3.3)	0.010
Anti-diabetic drugs, n (%)						
Metformin	14,910 (41.3)	5,404 (48.7)	0.149	4,273 (46.1)	4,190 (45.2)	0.018
Sulfonylureas	3,527 (9.8)	3,165 (28.5)	0.490	1,957 (21.1)	1,914 (20.7)	0.011
Alpha glucosidase inhibitors	44 (0.1)	74 (0.7)	0.087	23 (0.2)	19 (0.2)	0.009
DPP4i	1,598 (4.4)	1,881 (16.9)	0.414	995 (11.0)	972 (10.8)	0.008
SGLT2i	6,864 (19.0)	2,162 (19.5)	0.012	1,785 (19.8)	1,712 (19.0)	0.021
GLP1RA	17,976 (49.7)	1,934 (17.4)	0.729	1,975 (21.3)	1,908 (20.6)	0.018
Insulin	9,363 (25.9)	2,858 (25.7)	0.004	2,508 (27.1)	2,433 (26.3)	0.018
Type of TZDs, n (%)						
Pioglitazone	-	36,066 (99.9)	-	-	9,204 (99.4)	-
Rosiglitazone	-	71 (0.2)	-	-	58 (0.6)	-
Aspartate aminotransferase, U/L	29.5 ± 21.1	33.5 ± 38.3	0.130	30.7 ± 22.0	33.6 ± 40.5	0.088
Alanine aminotransferase, U/L	35.9 ± 29.0	40.1 ± 38.4	0.124	37.6 ± 30.2	40.2 ± 35.8	0.079
Platelet counts, 10^3^/uL	258 ± 79.0	241 ± 82.6	0.206	245 ± 77.0	242 ± 83.4	0.040
Hemoglobin A1c, %						
≥7	15,497 (42.9)	6,067 (54.6)	0.236	4,824 (52.1)	4,744 (51.2)	0.017
eGFR, mL/min/1.73m^2^						
<45	2,862 (7.9)	1,141 (10.3)	0.082	894 (9.7)	874 (9.4)	0.007
Systolic blood pressure, mmHg						
≥130	23,936 (66.2)	6,062 (54.6)	0.240	5,218 (56.3)	5,208 (56.2)	0.002
Cholesterol in HDL, mg/dL						
<50	17,791 (49.2)	4,682 (42.2)	0.142	3,927 (42.4)	3,874 (41.8)	0.012
Cholesterol in LDL, mg/dL						
≥130	4,426 (12.2)	985 (8.9)	0.110	806 (8.7)	841 (9.1)	0.013
Cholesterol, mg/dL						
≥200	6,449 (17.8)	1,751 (15.8)	0.056	1,409 (15.2)	1,453 (15.7)	0.013
Triglyceride, mg/dL						
≥150	13,893 (38.4)	3,890 (35.0)	0.071	3,327 (35.9)	3,226 (34.8)	0.023
Body mass index, kg/m^2^						
Mean (SD)	38.6 (8.0)	33.6 (7.5)	0.641	36.6 (7.5)	34.3 (7.5)	0.306
≥25	24,602 (68.1)	6,035 (54.3)	0.285	5,287 (57.1)	5,279 (57.0)	0.002
25–29.9	4,553 (12.6)	2,542 (22.8)	0.271	1,964 (21.2)	1,964 (21.2)	0.001
30–34.9	10,082 (27.9)	2,987 (26.8)	0.024	2,603 (28.1)	2,649 (28.6)	0.012
35–39.9	10,087 (30.1)	2,062 (18.5)	0.274	1,954 (21.1)	1,889 (20.4)	0.017
≥40	12,648 (35.0)	1,583 (14.2)	0.496	1,547 (16.7)	1,482 (16.0)	0.018

ACEi, angiotensin-converting enzyme inhibitor; ARB, angiotensin receptor blocker; DPP4i, dipeptidyl peptidase-4, inhibitor; eGFR, estimated glomerular filtration rate; GLP1-RA, glucagon-like peptide-1, receptor agonist; SGLT2i, sodium-glucose cotransporter-2, inhibitor; Std Diff, standardized difference; MASH, metabolic-dysfunction associated steatohepatitis; TZD, thiazolidinedione; TZP, tirzepatide

Standardized mean difference (SMD) < 0.1 is considered a small difference.

### Primary outcomes

**FIGURE 2:**
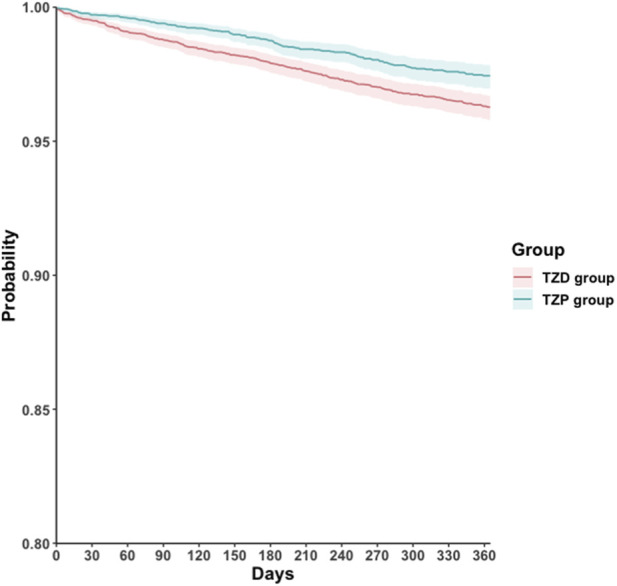
Kaplan-Meier time-to-event free curves of the composite outcome comparing the TZP and TZD groups. TZD, thiazolidinedione; TZP, tirzepatide.

During the one-year follow-up period, the incidence rate of the primary composite outcome was 1.8 per 100 person-years in the TZP group and 2.7 per 100 person-years in the TZD group. Accordingly, the TZP group was associated with a lower risk of the primary outcome compared to the TZD group (HR, 0.66; 95% CI, 0.54–0.82; E-value, 2.4; 95% LCL, 1.7) (Table 2). The Schoenfeld test indicated no violation of the proportional hazards assumption (p > 0.05). Survival analysis demonstrated a significantly higher cumulative probability of remaining free from the primary composite outcome in the TZP group compared to the TZD group (log-rank test, p < 0.001; Figure 2).

**TABLE 2:** Hazard ratio of outcomes between the TZP and the TZD groups.

Outcome	TZP group (n = 9,262)		TZD group (n = 9,262)		HR (95% CI)	*P* value	E-value (95% LCL)
	Events (n)	Incidence rate per 100 PYs	Events (n)	Incidence rate per 100 PYs			
Primary outcome							
Composite outcome	133	1.8	254	2.7	0.66 (0.54,0.82)	<0.001	2.4 (1.7)
Secondary outcomes							
All-cause mortality	34	0.5	91	1.0	0.48 (0.32,0.71)	<0.001	3.6 (2.2)
MACE	102	1.4	201	2.2	0.65 (0.51,0.82)	<0.001	2.5 (1.7)
MAKE	53	0.7	134	1.4	0.50 (0.36,0.69)	<0.001	3.4 (2.3)
MALO	69	0.9	136	1.5	0.64 (0.48,0.86)	0.003	2.5 (1.6)

CI, confidence interval; LCL, lower confidence limit; MACE, major adverse cardiovascular event; MAKE, major adverse kidney event; MALO, major adverse liver outcome; TZD, thiazolidinedione; TZP, tirzepatide

**FIGURE 3:**
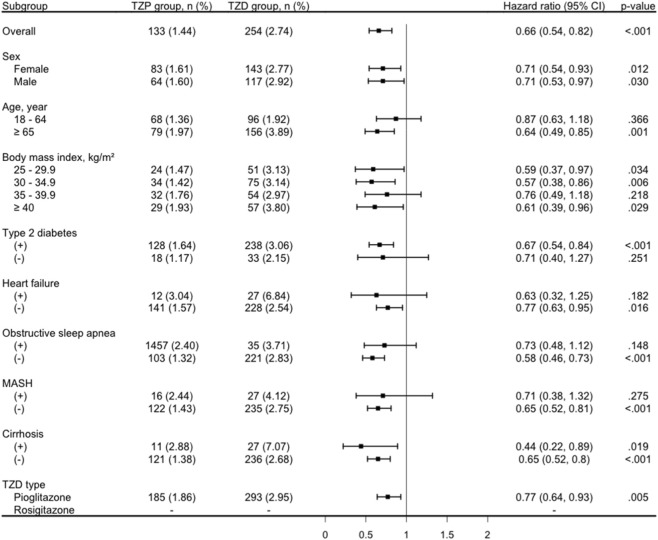
Stratified analysis of the composite outcome risk between the tirzepatide (TZP) and thiazolidinedione (TZD) groups. MASH, metabolic-dysfunction associated steatohepatitis.

Stratified analyses of the primary composite outcome showed consistent results, with HRs below 1 across most patient subgroups (Figure 3). In the sex-stratified analysis, HRs were 0.71 (95% CI, 0.54–0.93) in females and 0.71 (95% CI, 0.53–0.97) in males. Age-stratified analysis showed HRs of 0.87 (95% CI, 0.63–1.18) for patients aged 18–64 years and 0.64 (95% CI, 0.49–0.85) for those aged ≥65 years. BMI-stratified analysis demonstrated significantly reduced risks across most categories: HRs were 0.59 (95% CI, 0.37–0.97) for BMI 25–29.9 kg/m^2^, 0.57 (95% CI, 0.38–0.86) for BMI 30–34.9 kg/m^2^, 0.76 (95% CI, 0.49–1.18) for BMI 35–39.9 kg/m^2^, and 0.61 (95% CI, 0.39–0.96) for BMI ≥40 kg/m^2^. The TZP group also had significantly lower HR among patients with comorbid T2D (HR, 0.67; 95% CI, 0.54–0.84), without heart failure (HR, 0.77; 95% CI, 0.63–0.95), without OSA (HR, 0.58; 95% CI, 0.48-0.73), without MASH (HR, 0.65; 95% CI, 0.52–0.81), and without cirrhosis (HR, 0.65; 95% CI, 0.52–0.8). The results were consistent when comparing tirzepatide with pioglitazone, while separate analysis for rosiglitazone was not feasible due to the limited number of patients and events.

### Secondary outcomes

The use of TZP was associated with a significantly reduced risk of all-cause mortality (HR, 0.48; 95% CI, 0.32–0.71; E-value, 3.6; 95% LCL, 2.2), MACEs (HR, 0.65; 95% CI, 0.51–0.82; E-value, 2.5; 95% LCL, 1.7), MAKEs (HR, 0.50; 95% CI, 0.36–0.69; E-value, 3.4; 95% LCL, 2.3), and MALOs (HR, 0.64; 95% CI, 0.48–0.86; E-value, 2.5; 95% LCL, 1.6), as shown in Table 2.

### Additional analyses

To assess for potential residual confounding, three negative control outcomes were examined. There was no significant difference between groups in the incidence of hernia (HR, 0.89; 95% CI, 0.71–1.12; p = 0.317), hearing loss (HR, 1.08; 95% CI, 0.81–1.43; p = 0.603), or traumatic brain injury (HR, 1.25; 95% CI, 0.55–2.85; p = 0.593) (Supplementary Table 4). Landmark analysis from 1-month to 1-year follow-up showed consistent results for the primary composite outcome (HR, 0.73; 95% CI, 0.58–0.91; p = 0.005, Supplementary Table 5).

In a sensitivity analysis restricted to patients without prior GLP-1RA exposure before the index date, the direction of associations remained consistent with the primary analysis (Supplementary Table 6). Additional sensitivity analysis extending follow-up to 2 years, the results likewise remained consistent with the primary analysis (Supplementary Table 7). Regarding safety, TZP was not associated with higher risks of gastrointestinal adverse events, including nausea, vomiting, or diarrhea. In contrast, TZP users had significantly lower risks of edema, heart failure exacerbation, and fracture compared with TZD users (Supplementary Table 8). Lastly, we examined the individual components of the composite outcomes. TZP was associated with lower risks of stroke, dialysis, end-stage kidney disease, and ascites compared with thiazolidinedione therapy, while no significant differences were observed for the remaining components (Supplementary Table 9).

## Discussion

This retrospective study, which included over 18,000 individuals, is the first to compare the clinical effectiveness of TZP versus TZD in adults with MASLD. Our findings suggest that TZP is associated with improved clinical outcomes in this population. Specifically, TZP was associated with reductions of 52% in all-cause mortality, 35% in MACE, 50% in MAKE, and 36% in MALO. These associations remained consistent across most subgroups, including sex, age, BMI categories, and various comorbidities. These findings support the potential role of TZP as a preferred treatment option over TZD in adults with MASLD. Further prospective studies are warranted to confirm these benefits and inform clinical guidelines.

The superior effect of TZP compared to TZD observed in our study aligns with previous evidence. In a network meta-analysis (Souza et al., 2025) of 29 RCTs including 9,277 patients, TZP, along with pegozafermin and survodutide, was ranked among the most effective interventions, outperforming both vitamin E plus pioglitazone combination therapy and pioglitazone monotherapy in achieving MASH resolution without worsening fibrosis. In contrast, our study provides the first head-to-head comparison confirming the superiority of TZP for MASLD. Although both TZP and TZD are recommended for patients with MASLD (European Association for the Study of the Liver, 2024; American Diabetes Association Professional Practice Committee, 2025), our findings suggest that TZP may be associated with better clinical outcomes and could be considered the preferred therapeutic option in this clinical context.

The superior clinical benefits of TZP over TZD in MASLD can be attributed to multiple mechanisms supported by emerging scientific evidence. As a dual agonist of GIP and GLP-1 receptors, TZP induces potent and sustained weight loss, with clinical trials demonstrating mean reductions exceeding 25% of baseline body weight after prolonged treatment - substantially greater than those observed with placebo or other comparators (Aronne et al., 2024; Wadden et al., 2023; Aronne et al., 2025). This marked weight loss improves insulin sensitivity and reduces hepatic fat accumulation, directly benefiting liver health and slowing MASLD progression. Cardiovascular benefits are likely mediated through improvements in glycemic control, blood pressure, lipid profiles, and reductions in systemic inflammation, collectively lowering the risk of MACE and heart failure (Lin et al., 2023). TZP has been shown to slow kidney function decline and reduce albuminuria, potentially through hemodynamic and anti-inflammatory effects that mitigate kidney injury and progression to failure (Tian et al., 2025; Abasheva et al., 2024; Yang et al., 2025; Bosch et al., 2023). In parallel, TZP has demonstrated substantial benefits in patients with MASLD, including improvements in liver histology such as resolution of MASH and fibrosis without worsening disease severity (Loomba et al., 2024; Hu et al., 2025). These multifactorial effects on weight, metabolism, cardiovascular health, kidney function, and liver pathology provide a strong mechanistic rationale for the observed reductions in all-cause mortality, MALO, MACE, and MAKE in MASLD patients treated with TZP, underscoring its promise as a preferred therapeutic agent in this population.

The interpretation of our findings warrants careful consideration of the potential confounding role of prior GLP-1RA use. A substantial body of evidence has demonstrated that GLP-1RAs exert direct and indirect hepatoprotective effects in MASLD. Semaglutide has been shown to significantly reduce liver steatosis and improve histological activity in patients with MASH in a randomized trial (Sanyal et al., 2025), while liraglutide improved histological features of NASH and was associated with higher rates of resolution compared with placebo (Armstrong et al., 2016). These benefits extend beyond GLP-1RA monotherapy, with further evidence from meta-analyses and real-world studies confirming improvements in liver enzymes, hepatic fat content, and fibrosis-related markers (Ghosal et al., 2021; Ren et al., 2025). In our cohort, prior GLP-1RA use was substantially more prevalent in the TZP group before matching (49.7% vs. 17.4%), and despite achieving balance after PSM (∼21% in both groups), a meaningful proportion of patients in both arms had prior GLP-1RA exposure. Patients with such prior exposure may have already derived hepatic and metabolic benefits, including reductions in steatosis, inflammation, and cardiovascular risk, that could amplify the apparent advantage of TZP observed in the primary analysis. Indeed, when we restricted the analysis to patients without prior GLP-1RA use, effect estimates were more conservative, though the direction of all associations remained consistent (Supplementary Table 6). This pattern is consistent with the biological plausibility that residual hepatoprotective carry-over effects from prior GLP-1RA therapy partially contributed to the magnitude of TZP’s benefit in the primary cohort. Taken together, these findings underscore the importance of accounting for prior GLP-1RA exposure when interpreting comparative effectiveness studies of newer incretin-based therapies in MASLD.

This study has several notable strengths. Utilizing the TriNetX global network database allowed for the inclusion of a large, heterogeneous patient population, thereby improving statistical power and enhancing the generalizability of the results. The analysis of real-world clinical data adds to the translational relevance of the findings. Importantly, the adoption of an active comparator new-user design minimized biases related to treatment selection and temporal confounding, while PSM further ensured balanced baseline characteristics between the tirzepatide and comparator groups. Additionally, multiple sensitivity analyses, including landmark analysis and the use of negative control outcomes, were conducted to test the robustness of the findings under different assumptions and to assess potential residual confounding. Together, these comprehensive and rigorous methodological approaches strengthen the validity and clinical applicability of the observed associations.

Despite these strengths, several limitations should be acknowledged. First, although PSM effectively reduced confounding from measured covariates, BMI levels remained different between groups (Table 1). However, stratified analysis by BMI categories showed findings consistent with the primary analysis (Figure 3), supporting our results. Additionally, residual confounding from unmeasured factors, such as diet, exercise, and alcohol consumption, remains a limitation. Nevertheless, the high E-values observed indicate that any unmeasured confounder would need to be strongly associated with both the exposure and outcome to fully explain the observed associations, suggesting that the impact of residual confounding is likely limited. Second, detailed clinical information on MASLD severity, such as FIB-4, liver histology or fibrosis stage, was not available in the database, which may have led to disease misclassification or unmeasured heterogeneity in disease progression. To address this, subgroup analyses among individuals with MASH, or cirrhosis were performed and yielded results consistent with the primary findings. Third, the partially overlapping indications of TZP (approved for both obesity and T2D) and TZD (approved for T2D) represent a meaningful source of bias. Patients prescribed TZP are more likely to have a higher baseline BMI and a more obesity-driven phenotype, which may independently influence both liver-related and cardiovascular outcomes. Although PSM substantially reduced overt covariate imbalances and the high E-values support the robustness of our estimates, indication bias of this nature cannot be fully eliminated through statistical adjustment alone. Fourth, a potential temporal bias warrants consideration. Our study period begins in January 2022, so real-world adoption of TZP was likely concentrated toward the latter portion of the observation window. In contrast, TZD use is distributed more evenly across the study period, given its availability since the early 2000s. This temporal clustering may introduce differences in background standard-of-care practices, co-prescription patterns, and outcome ascertainment between groups. Although follow-up duration was analyzed as a time-to-event outcome to partially mitigate length-of-follow-up differences, and calendar-time adjusted analyses were considered, these were limited by sample size. Fifth, the statistical power for certain subgroup analyses, particularly in patients with comorbid OSA, was limited by the smaller number of patients and events within these strata. Although the direction of the association between TZP and the primary composite outcome among patients with OSA remained consistent with the overall analysis, the estimate did not reach statistical significance, and should therefore be interpreted with caution. This finding does not exclude a clinically meaningful benefit of TZP in this population; rather, it reflects the inherent limitation of subgroup analyses in observational cohorts, where sample sizes may be insufficient to detect modest effect differences with adequate precision. Adequately powered prospective studies specifically enrolling patients with MASLD and comorbid OSA are needed to clarify the therapeutic role of TZP in this subgroup. Lastly, the relatively short follow-up duration, particularly for the tirzepatide group given its initial FDA approval on 13 May 2022, may be insufficient to capture the full spectrum of long-term outcomes. Further studies are warranted to address these limitations.

## Conclusion

In this large real-world cohort study, TZP was associated with significantly better clinical outcomes compared to TZD in adults with MASLD, including substantial reductions in all-cause mortality, MALO, MACE, and MAKE. These benefits were consistent across diverse patient subgroups and supported by strong mechanistic rationale and robust sensitivity analyses. Given its multifaceted effects on metabolic, cardiovascular, renal, and hepatic pathways, TZP represents a promising therapeutic option for MASLD. Future prospective trials are needed to validate these findings and to inform evidence-based treatment guidelines.

## Data Availability

The original contributions presented in the study are included in the article/Supplementary Material, further inquiries can be directed to the corresponding author.

## References

[B1] American Diabetes Association Professional Practice Committee (2025). 4. Comprehensive medical evaluation and assessment of comorbidities: standards of care in Diabetes-2025. Diabetes Care 48 (Suppl. 1), S59–s85. 10.2337/dc25-S004 39651988 PMC11635044

[B2] Abasheva D. Ortiz A. Fernandez-Fernandez B. (2024). GLP-1 receptor agonists in patients with chronic kidney disease and either overweight or obesity. Clin. Kidney J. 17 (Suppl. 2), 19–35. 10.1093/ckj/sfae296 39583142 PMC11581768

[B3] Armstrong M. J. Gaunt P. Aithal G. P. Barton D. Hull D. Parker R. (2016). Liraglutide safety and efficacy in patients with non-alcoholic steatohepatitis (LEAN): a multicentre, double-blind, randomised, placebo-controlled phase 2 study. Lancet 387 (10019), 679–690. 10.1016/S0140-6736(15)00803-X 26608256

[B4] Aronne L. J. Sattar N. Horn D. B. Bays H. E. Wharton S. Lin W. Y. (2024). Continued treatment with tirzepatide for maintenance of weight reduction in adults with obesity: the SURMOUNT-4 randomized clinical trial. Jama 331 (1), 38–48. 10.1001/jama.2023.24945 38078870 PMC10714284

[B5] Aronne L. J. Horn D. B. le Roux C. W. Ho W. Falcon B. L. Gomez Valderas E. (2025). Tirzepatide as compared with semaglutide for the treatment of obesity. N. Engl. J. Med. 393 (1), 26–36. 10.1056/NEJMoa2416394 40353578

[B6] Bosch C. Carriazo S. Soler M. J. Ortiz A. Fernandez-Fernandez B. (2023). Tirzepatide and prevention of chronic kidney disease. Clin. Kidney J. 16 (5), 797–808. 10.1093/ckj/sfac274 37151412 PMC10157759

[B7] Chan K. E. Ong E. Y. H. Chung C. H. Ong C. E. Y. Koh B. Tan D. J. H. (2024). Longitudinal outcomes associated with metabolic dysfunction-associated steatotic liver disease: a meta-analysis of 129 studies. Clin. Gastroenterol. Hepatol. 22 (3), 488. 10.1016/j.cgh.2023.09.018 37775028

[B8] Ciardullo S. Mantovani A. Morieri M. L. Muraca E. Invernizzi P. Perseghin G. (2024). Impact of MASLD and MetALD on clinical outcomes: a meta-analysis of preliminary evidence. Liver Int. 44 (8), 1762–1767. 10.1111/liv.15939 38597738

[B9] Diaz L. A. Arab J. P. Idalsoaga F. Perelli J. Vega J. Dirchwolf M. (2025). Updated recommendations for the management of metabolic dysfunction-associated steatotic liver disease (MASLD) by the Latin American working group. Ann. Hepatol. 30, 101903. 10.1016/j.aohep.2025.101903 40089151

[B10] European Association for the Study of the Liver (EASL) (2024). EASL-EASD-EASO clinical practice guidelines on the management of metabolic dysfunction-associated steatotic liver disease (MASLD). J. Hepatol. 81(3):492–542. 10.1016/j.jhep.2024.04.031 38851997

[B11] Ghosal S. Datta D. Sinha B. (2021). A meta-analysis of the effects of glucagon-like-peptide 1 receptor agonist (GLP1-RA) in nonalcoholic fatty liver disease (NAFLD) with type 2 diabetes (T2D). Sci. Rep. 11 (1), 22063. 10.1038/s41598-021-01663-y 34764398 PMC8586228

[B12] Haukoos J. S. Lewis R. J. (2015). The propensity Score. Jama 314 (15), 1637–1638. 10.1001/jama.2015.13480 26501539 PMC4866501

[B13] Hu W. Gong W. Yang F. Cheng R. Zhang G. Gan L. (2025). Dual GIP and GLP-1 receptor agonist tirzepatide alleviates hepatic steatosis and modulates gut microbiota and bile acid metabolism in diabetic mice. Int. Immunopharmacol. 147, 113937. 10.1016/j.intimp.2024.113937 39752752

[B14] Jastreboff A. M. Aronne L. J. Ahmad N. N. Wharton S. Connery L. Alves B. (2022). Tirzepatide once weekly for the treatment of obesity. N. Engl. J. Med. 387 (3), 205–216. 10.1056/NEJMoa2206038 35658024

[B15] Karalliedde J. Buckingham R. E. (2007). Thiazolidinediones and their fluid-related adverse effects: facts, fiction and putative management strategies. Drug Saf. 30 (9), 741–753. 10.2165/00002018-200730090-00002 17722967

[B16] Kuo C. C. Chuang M. H. Li C. H. Tsai Y. W. Huang P. Y. Kuo H. T. (2025a). Glucagon-Like Peptide-1 receptor agonists and liver outcomes in patients with MASLD and type 2 diabetes. Aliment. Pharmacol. Ther. 61 (7), 1163–1174. 10.1111/apt.18502 39791391

[B17] Kuo C. C. Chuang M. H. Li C. H. Huang P. Y. Kuo H. T. Lai C. C. (2025b). Semaglutide and the risk of adverse liver outcomes in patients with nonalcoholic fatty liver disease and type 2 diabetes: a multi-institutional cohort study. Hepatol. Int. 19 (2), 395–404. 10.1007/s12072-024-10752-9 39602049

[B18] Lee M. Hong S. Cho Y. Rhee H. Yu M. H. Bae J. (2025). Synergistic benefit of thiazolidinedione and sodium-glucose cotransporter 2 inhibitor for metabolic dysfunction-associated steatotic liver disease in type 2 diabetes: a 24-week, open-label, randomized controlled trial. BMC Med. 23 (1), 266. 10.1186/s12916-025-04017-x 40336058 PMC12060414

[B19] Lin F. Yu B. Ling B. Lv G. Shang H. Zhao X. (2023). Weight loss efficiency and safety of tirzepatide: a systematic review. PLoS One 18 (5), e0285197. 10.1371/journal.pone.0285197 37141329 PMC10159347

[B20] Lin Y. M. Liao K. M. Yu T. Wu J. Y. Lai C. C. (2025). Effectiveness of tirzepatide in patients with HFpEF using a target trial emulation retrospective cohort study. Nat. Commun. 16 (1), 4471. 10.1038/s41467-025-59616-2 40368924 PMC12078458

[B21] Loomba R. Hartman M. L. Lawitz E. J. Vuppalanchi R. Boursier J. Bugianesi E. (2024). Tirzepatide for metabolic dysfunction-associated steatohepatitis with liver fibrosis. N. Engl. J. Med. 391 (4), 299–310. 10.1056/NEJMoa2401943 38856224

[B22] Mahady S. E. Webster A. C. Walker S. Sanyal A. George J. (2011). The role of thiazolidinediones in non-alcoholic steatohepatitis - a systematic review and meta analysis. J. Hepatol. 55 (6), 1383–1390. 10.1016/j.jhep.2011.03.016 21703200

[B23] Morgan C. J. (2019). Landmark analysis: a primer. J. Nucl. Cardiol. 26 (2), 391–393. 10.1007/s12350-019-01624-z 30719655

[B24] Musso G. Cassader M. Paschetta E. Gambino R. (2017). Thiazolidinediones and advanced liver fibrosis in nonalcoholic steatohepatitis: a meta-analysis. JAMA Intern Med. 177 (5), 633–640. 10.1001/jamainternmed.2016.9607 28241279 PMC5470366

[B25] Owrangi S. Paik J. M. Golabi P. de Avila L. Hashida R. Nader A. (2025). Meta-analysis: global prevalence and mortality of cirrhosis in metabolic dysfunction-associated steatotic liver disease. Aliment. Pharmacol. Ther. 61 (3), 433–443. 10.1111/apt.18451 39697043

[B26] Park M. J. Kim H. Kim M. G. Kim K. (2023). Comparison of glucagon-like peptide-1 receptor agonists and thiazolidinediones on treating nonalcoholic fatty liver disease: a network meta-analysis. Clin. Mol. Hepatol. 29 (3), 693–704. 10.3350/cmh.2022.0330 36907574 PMC10366812

[B27] Promrat K. Lutchman G. Uwaifo G. I. Freedman R. J. Soza A. Heller T. (2004). A pilot study of pioglitazone treatment for nonalcoholic steatohepatitis. Hepatology 39 (1), 188–196. 10.1002/hep.20012 14752837

[B28] Ratziu V. Giral P. Jacqueminet S. Charlotte F. Hartemann-Heurtier A. Serfaty L. (2008). Rosiglitazone for nonalcoholic steatohepatitis: one-year results of the randomized placebo-controlled fatty liver improvement with rosiglitazone therapy (FLIRT) trial. Gastroenterology 135 (1), 100–110. 10.1053/j.gastro.2008.03.078 18503774

[B29] Ren Q. Tan Y. Zhang G. Dai Y. Yang L. Wu Y. (2025). Efficacy of hypoglycemic agents in metabolic dysfunction associated steatotic liver disease (MASLD): a systematic review and network meta-analysis. J. Evid. Based Med. 18 (2), e70021. 10.1111/jebm.70021 40229658

[B30] Sanyal A. J. Newsome P. N. Kliers I. Østergaard L. H. Long M. T. Kjær M. S. (2025). Phase 3 trial of semaglutide in metabolic dysfunction-associated steatohepatitis. N. Engl. J. Med. 392 (21), 2089–2099. 10.1056/NEJMoa2413258 40305708

[B31] Souza M. Al-Sharif L. Antunes V. L. J. Huang D. Q. Loomba R. (2025). Comparison of pharmacological therapies in metabolic dysfunction-associated steatohepatitis for fibrosis regression and MASH resolution: systematic review and network meta-analysis. Hepatology. 82 (6), 1523–1533. 10.1097/HEP.0000000000001254 39903735 PMC12614381

[B32] Tang W. H. Francis G. S. Hoogwerf B. J. Young J. B. (2003). Fluid retention after initiation of thiazolidinedione therapy in diabetic patients with established chronic heart failure. J. Am. Coll. Cardiol. 41 (8), 1394–1398. 10.1016/s0735-1097(03)00159-1 12706937

[B33] Tian Y. Tian R. Juan H. Guo Y. Yan P. Cheng Y. (2025). GLP-1/GIP dual agonist tirzepatide normalizes diabetic nephropathy *via* PI3K/AKT mediated suppression of oxidative stress. Int. Immunopharmacol. 146, 113877. 10.1016/j.intimp.2024.113877 39700965

[B34] VanderWeele T. J. Ding P. (2017). Sensitivity analysis in observational research: introducing the E-Value. Ann. Intern Med. 167 (4), 268–274. 10.7326/M16-2607 28693043

[B35] Wadden T. A. Chao A. M. Machineni S. Kushner R. Ard J. Srivastava G. (2023). Tirzepatide after intensive lifestyle intervention in adults with overweight or obesity: the SURMOUNT-3 phase 3 trial. Nat. Med. 29 (11), 2909–2918. 10.1038/s41591-023-02597-w 37840095 PMC10667099

[B36] Wu J. Y. Chen C. C. Ling Tu W. Hsu W. H. Liu T. H. Tsai Y. W. (2025a). Clinical impact of tirzepatide on patients with OSA and obesity. Chest. 168 (3), 785–796. 10.1016/j.chest.2025.03.030 40254150

[B37] Wu J. Y. Chan S. E. Hsu W. H. Kuo C. C. Tsai Y. W. Liu T. H. (2025b). Comparing clinical outcomes of adults with obesity receiving tirzepatide versus bariatric metabolic surgery: a multi-institutional propensity score-matched study. Diabetes Obes. Metab. 27 (6), 3357–3366. 10.1111/dom.16353 40109063

[B38] Wu J. Y. Wu Y. J. Liu M. Y. Hsu W. H. Tsai Y. W. Liu T. H. (2025c). Clinical outcomes in diabetic patients with zinc deficiency: a multi-institutional population-based study. J. Am. Nutr. Assoc. 44, 1–8. 10.1080/27697061.2025.2461215 39908138

[B39] Yang Y. Wang Y. Zhou Y. Deng J. Wu L. (2025). Tirzepatide alleviates oxidative stress and inflammation in diabetic nephropathy via IL-17 signaling pathway. Mol. Cell. Biochem. 480 (2), 1241–1254. 10.1007/s11010-024-05066-1 38965127

[B40] Younossi Z. M. Kalligeros M. Henry L. (2025). Epidemiology of metabolic dysfunction-associated steatotic liver disease. Clin. Mol. Hepatol. 31 (Suppl. l), S32–s50. 10.3350/cmh.2024.0431 39159948 PMC11925440

[B41] Zhou B. G. Jiang X. She Q. Ding Y. B. (2024). Association of MASLD with the risk of extrahepatic cancers: a systematic review and meta-analysis of 18 cohort studies. Eur. J. Clin. Invest. 54 (11), e14276. 10.1111/eci.14276 38943276

