# Supplementary material for: Comparative effectiveness of tirzepatide versus thiazolidinedione in adults with MASLD: a propensity score-matched cohort study
Source: Front Pharmacol. 2026 May 15;17:1769425. doi: 10.3389/fphar.2026.1769425 (PMC13219383; doi:10.3389/fphar.2026.1769425)
Supplement: Supplementary file 1 [file DataSheet1.docx]

**eTable 1.** Demographic, diagnostic, procedural, medication, visit, and laboratory codes used in the definition of the cohorts

| **Category** | **Code** | **Description** |
| --- | --- | --- |
| **TZP group** | | |
| **#1**: At least 18 years old | | |
| Demographics | Age | Age (at least 18 years) |
| **#2**: Patients with MASLD treated with TZP  (# 2.1 must be fulfilled after #2.2) | | |
| **#2.1:** Patients with SGLT2i | | |
| Medication | NLM:RXNORM:2601723 | tirzepatide |
| **#2.2**: Patients with MASLD | | |
| Diagnosis | MASLD | [(1) OR (2)] AND  [(3) OR (4) OR (5) OR (6) OR (7) OR (8) OR (9) OR (10) OR (11) OR (12) OR (13) OR (14) OR (15) OR (16)] |
| Diagnosis | (1) UMLS: ICD-10-CM k76.0 | Fatty (change of) liver, not elsewhere classified |
| Diagnosis | (2) UMLS: ICD-10-CM k75.01 | Nonalcoholic steatohepatitis (NASH) |
| Labs | (3) LOINC 39156-5 | Body Mass Index ≥ 25 kg/m^2^ |
| Labs | (4) LOINC 8280-0 | Waist Circumference at umbilicus by Tape measure ≥ 94 cm |
| Labs | (5) LOINC 56115-9 | Waist Circumference by NCFS ≥ 94 cm |
| Labs | (6) TNX Curated 9037 | Hemoglobin Alc/ Hemoglobin. total in Blood ≥ 5.7% |
| Diagnosis | (7) IcD10cM E11 | Type 2 diabetes mellitus |
| Medication | (8) NLM:ATC A10A | insulins and analogues |
| Medication | (9) NLM:ATC A10B | blood glucose lowering drugs, excluding insulins |
| Labs | (10) LOINC 1558-6 | Fasting glucose [Mass/volume] in Serum or Plasma ≥ 100 mg/dL |
| Labs | (11) TNX Curated 9085 | Blood Pressure, Systolic ≥ 130 mmHg |
| Labs | (12) TNX Curated 9086 | Blood Pressure, Diastolic ≥ 85mmHg |
| Medication | (13) NLM:ATC C82 | Antihypertensives |
| Labs | (14) TNX Curated 9004 | Triglyceride [Mass/volume] in Serum, Plasma or Blood ≥ 150 mg/dL |
| Medication | (15) NLM:ATC C18 | lipid modifying agents |
| Labs | (16) TNX Curated 9001 | Cholesterol in HDL [Mass/volume] in Serum or Plasma ≥ 40mg/dL |
| **#3**: Without recent MACE, MAKE, or MALO (cannot have any of the following)  #3 must be fulfilled before #2.1 | | |
| Diagnosis | UMLS:ICD10CM:I21 | Acute myocardial infarction |
| Diagnosis | UMLS:ICD10CM:I46 | Cardiac arrest |
| Diagnosis | UMLS:ICD10CM:I22 | STEMI and NSTEMI |
| Diagnosis | UMLS:ICD10CM:I63 | Cerebral infarction |
| Diagnosis | UMLS:ICD10CM:Z99.2 | Dependence on renal dialysis |
| Diagnosis | UMLS:ICD10CM:N18.6 | End stage renal disease |
| Procedure | CPT:1012740 | Dialysis Services and Procedures |
| Lab | TNX:8001 | eGFR < 5 |
| Procedure | UMLS:ICD9CM:39.95 | Hemodialysis |
| Procedure | CPT:1012752 | Hemodialysis Procedures |
| Procedure | CPT:90945 | Dialysis procedure other than hemodialysis (eg, peritoneal dialysis, hemofiltration, or other continuous renal replacement therapies), with single evaluation by a physician or other qualified health care professional |
| Procedure | UMLS:CPT:1006747 | Hemodialysis Access, Intervascular Cannulation for Extracorporeal Circulation, or Shunt Insertion Procedures on Arteries and Veins |
| Diagnosis | UMLS:ICD10CM:I85.01 | Esophageal varices with bleeding |
| Diagnosis | UMLS:ICD10CM:I86.4 | Gastric varices |
| Diagnosis | UMLS:ICD10CM:K72 | Hepatic failure, not elsewhere classified |
| Diagnosis | UMLS:ICD10CM:K76.82 | Hepatic encephalopathy |
| Diagnosis | UMLS:ICD10CM:R18 | Ascites |
| Diagnosis | UMLS:ICD10CM:K65.2 | Spontaneous bacterial peritonitis |
| Diagnosis | UMLS:ICD10CM:K76.7 | Hepatorenal syndrome |
| Diagnosis | UMLS:ICD10CM:C22.0 | Liver cell carcinoma |
| Procedure | UMLS:ICD10PCS:0FY0 | Hepatobiliary System And Pancreas / Transplantation / Liver |
| Diagnosis | UMLS:ICD10PCS:Z94.4 | Liver transplant status |
| **#4**: Without combination of study medications (cannot have at the same time) | | |
| medication | NLM:RXNORM:2601723 | tirzepatide |
| medication | NLM:ATC:A10BG | Thiazolidinediones |
| **#5**: Incident user (cannot have any of the following)  #5 must be fulfilled before #2.1 | | |
| medication | NLM:RXNORM:2601723 | tirzepatide |
| medication | NLM:ATC:A10BG | Thiazolidinediones |
| **#6**: Have a follow-up record (have any of the following)  #6 must be fulfilled within 1 day and 1 year after #2.1 | | |
| Visit | Deceased | Deceased |
| Diagnosis | UMLS:ICD10CM:R99 | Ill-defined and unknown cause of mortality |
| Visit | Visit | Visit |
| **#7**: Without T1D | | |
| Diagnosis | UMLS: ICD-10-CM E10 | Type 1 diabetes mellitus |
| **#8**: Without chronic liver events (cannot have any of the following) | | |
| Diagnosis | UMLS: ICD-10-CM K70 | Alcoholic liver disease |
| Diagnosis | UMLS: ICD-10-CM K73 | Chronic hepatitis, not elsewhere classified |
| Diagnosis | UMLS: ICD-10-CM K75.4 | Autoimmune hepatitis |
| Diagnosis | UMLS: ICD-10-CM B15-B19 | Viral hepatitis |
| Diagnosis | UMLS: ICD-10-CM K71 | Toxic liver disease |
| Diagnosis | UMLS: ICD-10-CM K75.0 | Abscess of liver |
| Diagnosis | UMLS: ICD-10-CM K75.2 | Nonspecific reactive hepatitis |
| Diagnosis | UMLS: ICD-10-CM K75.3 | Granulomatous hepatitis, not elsewhere classified |
| Diagnosis | UMLS: ICD-10-CM HZ2 | Detoxification Services |
| Diagnosis | UMLS: ICD-10-CM I82.0 | Budd-Chiari syndrome |
| Diagnosis | UMLS: ICD-10-CM K83.01 | Primary sclerosing cholangitis |
| Diagnosis | UMLS: ICD-10-CM E83.01 | Wilson's disease |
| **#9**: Visit HCOs twice since 2022 | | |
| Visit | Visit | Visit more than twice since 2022 |
| **DPP4i group** | | |
| **#1**: At least 18 years old | | |
| Demographics | Age | Age (at least 18 years) |
| **#2**: Patients with MASLD treated with TZD  (# 2.1 must be fulfilled after #2.2) | | |
| **#2.1**: Patients with DPP4i | | |
| medication | NLM:ATC:A10BG | Thiazolidinediones |
| **#2.2**: Patients with MASLD | | |
| Diagnosis | MASLD | [(1) OR (2)] AND  [(3) OR (4) OR (5) OR (6) OR (7) OR (8) OR (9) OR (10) OR (11) OR (12) OR (13) OR (14) OR (15) OR (16)] |
| Diagnosis | (1) UMLS: ICD-10-CM k76.0 | Fatty (change of) liver, not elsewhere classified |
| Diagnosis | (2) UMLS: ICD-10-CM k75.01 | Nonalcoholic steatohepatitis (NASH) |
| Labs | (3) LOINC 39156-5 | Body Mass Index ≥ 25 kg/m^2^ |
| Labs | (4) LOINC 8280-0 | Waist Circumference at umbilicus by Tape measure ≥ 94 cm |
| Labs | (5) LOINC 56115-9 | Waist Circumference by NCFS ≥ 94 cm |
| Labs | (6) TNX Curated 9037 | Hemoglobin Alc/ Hemoglobin. total in Blood ≥ 5.7% |
| Diagnosis | (7) IcD10cM E11 | Type 2 diabetes mellitus |
| Medication | (8) NLM:ATC A10A | insulins and analogues |
| Medication | (9) NLM:ATC A10B | blood glucose lowering drugs, excluding insulins |
| Labs | (10) LOINC 1558-6 | Fasting glucose [Mass/volume] in Serum or Plasma ≥ 100 mg/dL |
| Labs | (11) TNX Curated 9085 | Blood Pressure, Systolic ≥ 130 mmHg |
| Labs | (12) TNX Curated 9086 | Blood Pressure, Diastolic ≥ 85mmHg |
| Medication | (13) NLM:ATC C82 | Antihypertensives |
| Labs | (14) TNX Curated 9004 | Triglyceride [Mass/volume] in Serum, Plasma or Blood ≥ 150 mg/dL |
| Medication | (15) NLM:ATC C18 | lipid modifying agents |
| Labs | (16) TNX Curated 9001 | Cholesterol in HDL [Mass/volume] in Serum or Plasma ≥ 40mg/dL |
| **#3**: Without recent MACE, MAKE, or MALO (cannot have any of the following)  #3 must be fulfilled before #2.1 | | |
| Diagnosis | UMLS:ICD10CM:I21 | Acute myocardial infarction |
| Diagnosis | UMLS:ICD10CM:I46 | Cardiac arrest |
| Diagnosis | UMLS:ICD10CM:I22 | STEMI and NSTEMI |
| Diagnosis | UMLS:ICD10CM:I63 | Cerebral infarction |
| Diagnosis | UMLS:ICD10CM:Z99.2 | Dependence on renal dialysis |
| Diagnosis | UMLS:ICD10CM:N18.6 | End stage renal disease |
| Procedure | CPT:1012740 | Dialysis Services and Procedures |
| Lab | TNX:8001 | eGFR < 5 |
| Procedure | UMLS:ICD9CM:39.95 | Hemodialysis |
| Procedure | CPT:1012752 | Hemodialysis Procedures |
| Procedure | CPT:90945 | Dialysis procedure other than hemodialysis (eg, peritoneal dialysis, hemofiltration, or other continuous renal replacement therapies), with single evaluation by a physician or other qualified health care professional |
| Procedure | UMLS:CPT:1006747 | Hemodialysis Access, Intervascular Cannulation for Extracorporeal Circulation, or Shunt Insertion Procedures on Arteries and Veins |
| Diagnosis | UMLS:ICD10CM:I85.01 | Esophageal varices with bleeding |
| Diagnosis | UMLS:ICD10CM:I86.4 | Gastric varices |
| Diagnosis | UMLS:ICD10CM:K72 | Hepatic failure, not elsewhere classified |
| Diagnosis | UMLS:ICD10CM:K76.82 | Hepatic encephalopathy |
| Diagnosis | UMLS:ICD10CM:R18 | Ascites |
| Diagnosis | UMLS:ICD10CM:K65.2 | Spontaneous bacterial peritonitis |
| Diagnosis | UMLS:ICD10CM:K76.7 | Hepatorenal syndrome |
| Diagnosis | UMLS:ICD10CM:C22.0 | Liver cell carcinoma |
| Procedure | UMLS:ICD10PCS:0FY0 | Hepatobiliary System And Pancreas / Transplantation / Liver |
| Diagnosis | UMLS:ICD10PCS:Z94.4 | Liver transplant status |
| **#4**: Without combination of study medications (cannot have at the same time) | | |
| medication | NLM:RXNORM:2601723 | tirzepatide |
| medication | NLM:ATC:A10BG | Thiazolidinediones |
| **#5**: Incident user (cannot have any of the following)  #5 must be fulfilled before #2.1 | | |
| medication | NLM:RXNORM:2601723 | tirzepatide |
| medication | NLM:ATC:A10BG | Thiazolidinediones |
| **#6**: Have a follow-up record (have any of the following)  #6 must be fulfilled within 1 day and 1 year after #2.1 | | |
| Visit | Deceased | Deceased |
| Diagnosis | UMLS:ICD10CM:R99 | Ill-defined and unknown cause of mortality |
| Visit | Visit | Visit |
| **#7**: Without T1D | | |
| Diagnosis | UMLS: ICD-10-CM E10 | Type 1 diabetes mellitus |
| **#8**: Without chronic liver events (cannot have any of the following) | | |
| Diagnosis | UMLS: ICD-10-CM K70 | Alcoholic liver disease |
| Diagnosis | UMLS: ICD-10-CM K73 | Chronic hepatitis, not elsewhere classified |
| Diagnosis | UMLS: ICD-10-CM K75.4 | Autoimmune hepatitis |
| Diagnosis | UMLS: ICD-10-CM B15-B19 | Viral hepatitis |
| Diagnosis | UMLS: ICD-10-CM K71 | Toxic liver disease |
| Diagnosis | UMLS: ICD-10-CM K75.0 | Abscess of liver |
| Diagnosis | UMLS: ICD-10-CM K75.2 | Nonspecific reactive hepatitis |
| Diagnosis | UMLS: ICD-10-CM K75.3 | Granulomatous hepatitis, not elsewhere classified |
| Diagnosis | UMLS: ICD-10-CM HZ2 | Detoxification Services |
| Diagnosis | UMLS: ICD-10-CM I82.0 | Budd-Chiari syndrome |
| Diagnosis | UMLS: ICD-10-CM K83.01 | Primary sclerosing cholangitis |
| Diagnosis | UMLS: ICD-10-CM E83.01 | Wilson's disease |
| **#9**: Visit HCOs twice since 2022 | | |
| Visit | Visit | Visit more than twice since 2022 |

**eTable 2.** Demographic, diagnostic, and laboratory codes used in the definition of covariates

| **Category** | **Code** | **Description** |
| --- | --- | --- |
| Demographics | AI | Age at index |
| Demographics | F | Female |
| Demographics | 2106-3 | White |
| Demographics | UNK | Unknown Race |
| Demographics | 2054-5 | Black or African American |
| Demographics | 2028-9 | Asian |
| Demographics | 2131-1 | Other Race |
| Lab | 9083 | BMI |
| Lab | 9037 | Hemoglobin A1c |
| Lab | 9002 | Cholesterol in LDL |
| Lab | 9000 | Cholesterol |
| Lab | 9001 | Cholesterol in HDL |
| Lab | 9004 | Triglyceride |
| Lab | 8001 | eGFR |
| Lab | 9085 | Blood Pressure, Systolic |
| Medication | C10AA | HMG CoA reductase inhibitors |
| Medication | CV100 | BETA BLOCKERS/RELATED |
| Medication | CV200 | CALCIUM CHANNEL BLOCKERS |
| Medication | CV700 | DIURETICS |
| Medication | CV800 | ACE INHIBITORS |
| Medication | CV805 | ANGIOTENSIN II INHIBITOR |
| Medication | A10A | INSULINS AND ANALOGUES |
| Medication | A10BA | Biguanides |
| Medication | A10BB | Sulfonylureas |
| Medication | A10BF | Alpha glucosidase inhibitors |
| Medication | A10BH | DPP4i |
| Medication | A10BK | SGLT2i |
| Medication | A10BJ | Glucagon-like peptide-1 (GLP-1) analogues |
| Medication | 341248 | ezetimibe |
| Medication | C10AB | Fibrates |
| Diagnosis | E11 | Type 2 diabetes mellitus |
| Diagnosis | E11.2 | Type 2 diabetes mellitus with kidney complications |
| Diagnosis | E11.3 | Type 2 diabetes mellitus with ophthalmic complications |
| Diagnosis | E11.4 | Type 2 diabetes mellitus with neurological complications |
| Diagnosis | E11.5 | Type 2 diabetes mellitus with circulatory complications |
| Diagnosis | K75.81 | Nonalcoholic steatohepatitis (NASH) |
| Diagnosis | K76.0 | Fatty (change of) liver, not elsewhere classified |
| Diagnosis | K74.0 | Hepatic fibrosis |
| Diagnosis | K74.6 | Other and unspecified cirrhosis of liver |
| Diagnosis | C00-D49 | Neoplasms |
| Diagnosis | J40-J4A | Chronic lower respiratory diseases |
| Diagnosis | F10 | Alcohol related disorders |
| Diagnosis | F17 | Nicotine dependence |
| Diagnosis | E66 | Overweight and obesity |
| Diagnosis | I10 | Essential (primary) hypertension |
| Diagnosis | E78 | Disorders of lipoprotein metabolism and other lipidemias |
| Diagnosis | I48 | Atrial fibrillation and flutter |
| Diagnosis | I50 | Heart failure |
| Diagnosis | I60-I69 | Cerebrovascular diseases |
| Diagnosis | I20-I25 | Ischemic heart diseases |
| Diagnosis | N18 | Chronic kidney disease (CKD) |

**eTable 3.** Diagnostic, visit, and procedural codes used in the definition of outcomes

| **Category** | **Code** | **Description** |
| --- | --- | --- |
| **#1**: All-cause mortality (have any of the following) | | |
| Visit | Deceased | Deceased |
| Diagnosis | UMLS:ICD10CM:R99 | Ill-defined and unknown cause of mortality |
| **#2**: MACE (have any of the following)  Exclude patients with outcomes prior to the index date | | |
| Demographics | Deceased | Deceased |
| Diagnosis | UMLS:ICD10CM:R99 | Ill-defined and unknown cause of mortality |
| Diagnosis | UMLS:ICD10CM:I21 | Acute myocardial infarction |
| Diagnosis | UMLS:ICD10CM:I22 | Subsequent ST elevation (STEMI) and non-ST elevation (NSTEMI) myocardial infarction |
| Diagnosis | UMLS:ICD10CM:I63 | Cerebral infarction |
| Diagnosis | UMLS:ICD10CM:I46 | Cardiac arrest |
| **#3**: MAKE (have any of the following)  Exclude patients with outcomes prior to the index date | | |
| Demographics | Deceased | Deceased |
| Diagnosis | UMLS:ICD10CM:R99 | Ill-defined and unknown cause of mortality |
| Procedure | UMLS:CPT:90945 | Dialysis procedure other than hemodialysis (eg, peritoneal dialysis, hemofiltration, or other continuous renal replacement therapies), with single evaluation by a physician or other qualified health care professional |
| Procedure | UMLS:CPT:1012740 | Dialysis Services and Procedures |
| Diagnosis | UMLS:ICD10CM:Z99.2 | Dependence on renal dialysis |
| Procedure | UMLS:ICD9CM:39.95 | Hemodialysis |
| Procedure | UMLS:CPT:1006747 | Hemodialysis Access, Intervascular Cannulation for Extracorporeal Circulation, or Shunt Insertion Procedures on Arteries and Veins |
| Procedure | UMLS:CPT:1012752 | Hemodialysis Procedures |
| Diagnosis | UMLS:ICD10CM:N18.6 | End stage renal disease |
| Laboratory | TNX:8001 | Glomerular filtration rate/1.73 sq M.predicted [Volume Rate/Area] in Serum, Plasma or Blood by Creatinine-based formula (MDRD) (at most 5.00 mL/min/{1.73_m2} (most recent occurrence)) |
| #4: MALO (have any of the following)  Exclude patients with outcomes prior to the index date | | |
| Diagnosis | UMLS:ICD10CM:I85.01 | Esophageal varices with bleeding |
| Diagnosis | UMLS:ICD10CM:I86.4 | Gastric varices |
| Diagnosis | UMLS:ICD10CM:K72 | Hepatic failure, not elsewhere classified |
| Diagnosis | UMLS:ICD10CM:K76.82 | Hepatic encephalopathy |
| Diagnosis | UMLS:ICD10CM:R18 | Ascites |
| Diagnosis | UMLS:ICD10CM:K65.2 | Spontaneous bacterial peritonitis |
| Diagnosis | UMLS:ICD10CM:K76.7 | Hepatorenal syndrome |
| Diagnosis | UMLS:ICD10CM:C22.0 | Liver cell carcinoma |
| Procedure | UMLS:ICD10PCS:0FY0 | Hepatobiliary System And Pancreas / Transplantation / Liver |
| Diagnosis | UMLS:ICD10CM:Z94.4 | Liver transplant status |
| Demographics | Deceased | Deceased |
| Diagnosis | UMLS:ICD10CM:R99 | Ill-defined and unknown cause of mortality |
| #5 Composite endpoint  Composite of #1~#4 | | |

**eTable 4**. Negative outcomes control between the TZP group and the TZD group.

| Outcome | HR (95% CI) | *P* value |
| --- | --- | --- |
|  |  |  |
| Hernia | 0.89 (0.71,1.12) | 0.317 |
| Hearing loss | 1.08 (0.81,1.43) | 0.603 |
| Traumatic brain injury | 1.25 (0.55,2.85) | 0.593 |

**eTable 5**. Sensitivity analysis with Landmark analysis of the primary outcome.

| Outcome | HR (95% CI) | *P* value |
| --- | --- | --- |
|  |  |  |
| 1-month to 1-year |  |  |
| Composite outcome | 0.73 (0.58,0.91) | 0.005 |

**eTable 6**. Sensitivity analysis restricted to patients without prior GLP-1RA use before the index date.

| Outcome | HR (95% CI) | *P* value |
| --- | --- | --- |
|  |  |  |
| Composite outcome | 0.78 (0.68,0.90) | <.001 |
| All-cause mortality | 0.39 (0.25,0.59) | <.001 |
| MACE | 0.80 (0.62,1.02) | 0.071 |
| MAKE | 0.58 (0.42,0.81) | <.001 |
| MALO | 0.63 (0.46,0.86) | 0.003 |

**eTable 7**. Sensitivity analysis with extended follow-up to 2 years.

| Outcome | HR (95% CI) | *P* value |
| --- | --- | --- |
|  |  |  |
| Composite outcome | 0.83 (0.71,0.97) | 0.019 |
| All-cause mortality | 0.62 (0.47,0.82) | <.001 |
| MACE | 0.82 (0.69,0.98) | 0.024 |
| MAKE | 0.71 (0.57,0.89) | 0.003 |
| MALO | 0.74 (0.60,0.91) | 0.005 |

**eTable 8.** Safety outcomes.

| Outcome | HR (95% CI) | *P* value |
| --- | --- | --- |
|  |  |  |
| Nausea | 1.07 (0.93,1.23) | 0.359 |
| Vomiting | 1.19 (0.91,1.56) | 0.208 |
| Diarrhea | 0.97 (0.85,1.11) | 0.671 |
| Edema | 0.82 (0.72,0.94) | 0.003 |
| Heart failure exacerbation | 0.51 (0.42,0.63) | <.001 |
| Fracture | 0.75 (0.62,0.91) | 0.004 |
| Hypoglycemia | 0.85 (0.60,1.18) | 0.327 |

**eTable 9**. Sensitivity analysis using individual components of outcomes.

| Outcome | HR (95% CI) | *P* value |
| --- | --- | --- |
|  |  |  |
| Acute myocardial infarction | 0.94 (0.74,1.18) | 0.582 |
| Stroke | 0.80 (0.66,0.97) | 0.025 |
| Cardiac arrest | 0.63 (0.30,1.30) | 0.205 |
| Dialysis services | 0.56 (0.31,1.00) | 0.049 |
| End stage kidney disease | 0.50 (0.38,0.65) | <.001 |
| Hepatic encephalopathy | 0.97 (0.49,1.91) | 0.926 |
| Ascites | 0.52 (0.35,0.78) | 0.014 |
